# Permanent Pacing Reduces Blood Pressure in Older Patients with Drug-resistant Hypertension: A New Pacing Paradigm?

**DOI:** 10.19102/icrm.2024.15091

**Published:** 2024-09-15

**Authors:** Bich Lien Nguyen, Michael H. Burnam, Francesco Accardo, Angela Angione, Roberto Scacciavillani, Carly Pierson, Eli S. Gang

**Affiliations:** 1 Cardiology Department, Sapienza University, Rome, Italy; 2 BaroPace, Ashland, OR, USA; 3 Providence Cedars-Sinai Tarzana Medical Center, Tarzana, CA, USA; 4 Agostino Gemelli University, Rome, Italy; 5 Cardiovascular Research Foundation, Beverly Hills, CA, USA; 6 Heart Institute, Cedars-Sinai Medical Center, Los Angeles, CA, USA

**Keywords:** Drug-resistant hypertension, dual-chamber pacing, hypertension, permanent pacing

## Abstract

Hypertension (HTN) is a major contributor to cardiovascular mortality. Many patients with drug-resistant hypertension (DRH) also require permanent pacing (PP). This large retrospective study evaluated the effect of PP for conventional PP indications in older patients with DRH. We reviewed the charts of 176 patients with dual-chamber PP and DRH. The effects of PP on systolic and diastolic blood pressure (sBP and dBP), the number of HTN-related medications, and left ventricular ejection fraction (LVEF) were assessed at 6 months post-implantation and compared with pre-implantation values. Patients were followed up with for ≥72 months. Patients with a decline of >5 mmHg in sBP and decrease in at least one anti-HTN medication were defined as responders (126/176; *P* < .01). The mean decline in sBP was 9 mmHg, while that in dBP was 3 mmHg (*P* < .001 for both). Among responders, optimal reductions in sBP, dBP, and medications were seen at a stratification of >50% atrial pacing and <40% ventricular pacing (−12, −6.3, and −1.6, respectively). When right ventricular pacing of <50% was used for dichotomizing, the optimal atrial/ventricular pacing stratification was atrial pacing > 50% and ventricular pacing < 40% (−11.3, −6.3, and −1.6, respectively). A relationship between increasing atrial pacing and a decline in sBP was noted but did not reach statistical significance. However, of those responders who had a >10-mmHg decline in sBP, the majority were paced between 60%–100% in the atria. The LVEF did not change post-PP in either group. In conclusion, PP results in significant improvement in BP control. The observed association warrants further investigation.

## Introduction

Drug-resistant hypertension (DRH) is defined as blood pressure (BP) that remains above the goal despite concomitant use of at least three different classes of antihypertensive drugs, administered at maximally tolerated doses, including a diuretic.^1,2^ Patients with DRH are at high risk of experiencing major cardiovascular events.^3,4^ The prevalence and incidence of DRH are expected to increase as the global population continues to age, with an absolute increase in the number of affected individuals as the general population grows.^5^

Recent efforts to address the problem of DRH include the development and investigation of device-based therapies. Because many patients have high resting sympathetic activity,^1,2,6,7^ one focus in the past decade has been the development of several implantable devices, including those intended to target the autonomic nervous system, regulate left ventricular preload, or alter mechanical arterial properties. Non-pharmacological neuromodulation devices that modulate sympathetic activity using electrical activation of the carotid baroreflex, catheter-based renal nerve ablation, and new algorithms for permanent ventricular pacing are supported by experimental studies and early clinical trials.^8–17^ Research has also begun to support device-based hypertension (HTN) control using baroreceptor stimulation^8^ and atrioventricular (AV) delay modulation^9^ in patients with permanently implanted pacemakers.

However, the relationship between HTN and conventional clinical permanent cardiac pacing is not well established. Many older patients requiring permanent pacing (PP) also have persistent HTN with systolic blood pressure (sBP) values above the recommended levels. We previously observed a significant reduction in sBP and diastolic BP (dBP) among such patients,^18^ and a randomized multicenter clinical trial in patients with HTN and heart failure with preserved ejection fraction is currently underway (ClinicalTrials.gov identifier no. NCT06036186). In this retrospective study, we sought to evaluate the effect of permanent cardiac pacing in a large group of elderly patients with DRH and to provide possible pathophysiological insights into pacing-mediated BP reduction.

## Methods

### Patient population

We performed a retrospective review of charts of patients who had undergone implantation of a permanent dual-chamber pacemaker for standard clinical indications during the period between May 2012 and June 2022. Patients also needed to have documentation of DRH, ie, HTN requiring at least 3 anti-HTN medications, with one of them usually being a diuretic.^1,2^ Medications had been given at standard clinical practice doses. Patients with persistent atrial fibrillation (AF) were excluded from the study. Standard demographic data were collected, and pre- and post-implant left ventricular ejection fractions (LVEFs) were recorded. Each patient’s list of medications was reviewed and compared before and after the initiation of PP. The initial BP measurement was the closest one obtained prior to pacemaker implantation and measured the day prior to the procedure. Medications were not held on the day of the procedure. The 6-month follow-up evaluation and resting BP cuff measurement at that visit were used for determining whether a patient was a “responder” or a “non-responder” to PP (see the Definition section). The BP and medication data from subsequent clinic visits were also collected, but not used in the analysis. Patients were managed in the pacemaker clinic by a cardiologist. Pacemaker patients were followed up with every 6–12 months, similar to the other non-pacemaker patients. The study was approved by the appropriate institutional review board, and written informed consent was obtained.

### Definition

Patients were defined at the 6-month follow-up visit as responders to PP if they manifested an sBP decline of ≥5 mmHg or had reduced their anti-HTN medication regimen by at least one medication(s).^8^

### Statistical analyses

The demographic table **(Table 1)** is stratified by “responder” and “non-responder” groups. For categorical variables, percentages are reported for each group, and *P* values of Fisher’s exact tests for comparison between the response groups are given. For continuous variables, mean and standard deviation values are reported for each group, and *P* values of Wilcoxon rank-sum tests for comparison between the response groups are given. The *P* value of the Wald test is reported to compare the proportion of defined “responders” (126/176) to 5%. The *P* value of the paired *t* test is reported to compare the number of baseline medications to the number of post-implant medications for the defined groups. A linear regression line is fitted in each scatterplot to show the correlation between ventricular pacing/atrial pacing and BPs (sBP and dBP) for the defined “responder.” Wilcoxon signed-rank tests were used to compare the change in BP (between baseline and post-pacing) for the two response groups. For the relevant tests, a *P* < .01 was considered to be statistically significant.

## Results

### Description of study patients

As shown in **Table 1**, the demographic characteristics of the two groups, responders and non-responders, were similar with respect to age, sex distribution, and pacing indications. As discussed in greater detail later, the atrium was paced more frequently among responders than non-responders. Responders as a group also had a higher pre-PP sBP values. Meanwhile, the distribution of HTN medications between the two groups was similar, with the exception of calcium channel blockers, which were more prevalent among the responders. Patients who had a longer follow-up **(Table 1)** remained either responders or non-responders, ie, unchanged from their group designation at 6 months. Thus, while the assignment to a response group was made at the 6-month visit, the actual follow-up period was significantly longer for most patients. The mean AF burden detected by devices was 14.2% ± 9.7% and did not differ between groups (*P* > .05).

### Responders versus non-responders

Using the described definition, 126 patients were considered as responders and 50 were considered as non-responders. Using the Wald test, the proportion of responders (126/176) was significantly different from a random 5% occurrence, with *P* < .001.

**Figure 1:**
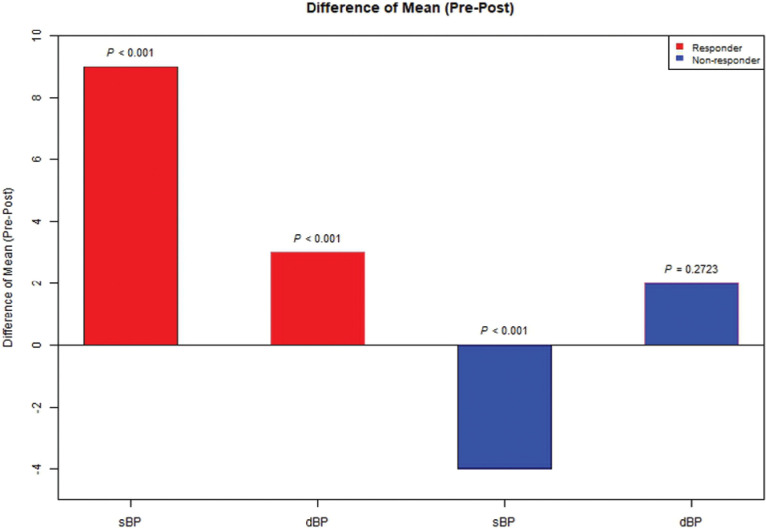
Differences in mean blood pressure (pre-/post-pacing). A “bar plot” illustrating mean blood pressure (before and after permanent pacing) in both groups of patients (responders and non-responders). *Abbreviations:* dBP, diastolic blood pressure; sBP, systolic blood pressure.

As shown in **Table 2**, the mean differences in sBP and dBP before and after PP among responders were highly significant, with a decrease from 130 ± 9.8 to 121 ± 10.2 mmHg (sBP), with a mean drop of 9 mmHg (*P* < .001), and a decrease from 79 ± 5.8 to 76 ± 7.5 mmHg (dBP), with a mean drop of 3 mmHg (*P* < .001). Conversely, among the non-responders, the sBP actually rose by 4 mmHg, while the observed dBP decline of 2 mmHg did not reach statistical significance. The bar plot in **Figure 1** graphically displays these data.

### Distribution of systolic blood pressure measurements among responders

**Figure 2:**
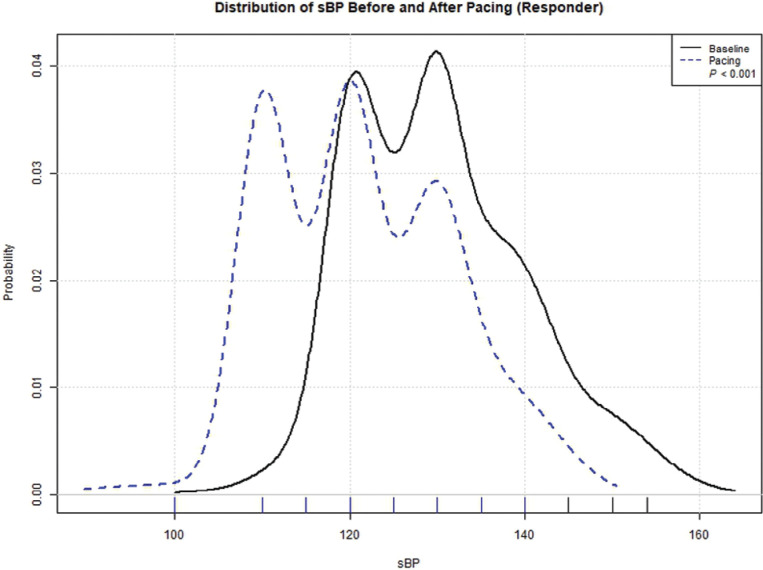
Distribution of systolic blood pressure (sBP) (mmHg) before and after pacing. A Wilcoxon signed-rank test was used for comparing the distribution of sBP among responders (n = 126) before and after the initiation of permanent pacing (*P* < .001). Note the significant “shift to the left” of the sBP curve (dotted line) following pacing among the responders. *Abbreviation:* sBP, systolic blood pressure.

As shown in **Figure 2**, the sBP distribution curve among responders was shown to significantly shift to the left, ie, toward lower sBP values, following the initiation of PP, with a *P* < .001 result using the Wilcoxon signed-rank test.

### Relationship between response to pacing, decline in systolic blood pressure, and change in the number of medications

**Figure 3:**
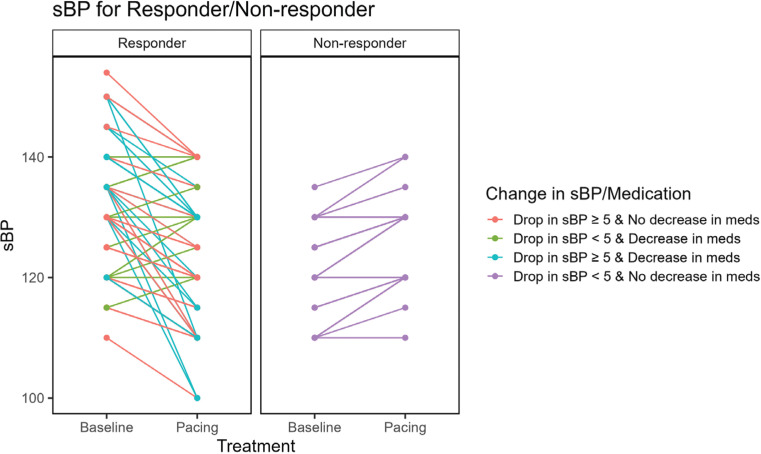
Relationship between pacing, decline in systolic blood pressure (sBP), and changes in the number of medications. A majority of the responders had either a decline in sBP of ≥5 mmHg and a reduction by at least one medication or a decline in sBP without a change in medication. Only a small number of responders (n = 8) had a reduction in medication number without a decline in sBP of ≥5 mmHg. Among the 50 non-responders, 9 patients had an increase in the number of medications taken after permanent pacing. The number of lines in the graph does not correspond to all the patients, as many patients who had similar responses are grouped into single graph lines for the sake of clarity. *Abbreviation:* sBP, systolic blood pressure.

**Figure 3** graphically displays the relationship between response to PP as it applied to a decline in sBP and/or to changes in the number of HTN medications taken. The majority of responders experienced either a decline in sBP of ≥5 mmHg and a decline by at least one medication or a decline in sBP without a change in medication. Only a small number of responders (n = 8) had a reduction in medication number without a decline in sBP of ≥5 mmHg. Among the 50 non-responders, 9 patients actually had an increase in the number of medications taken post-PP. The number of lines in the graph in **Figure 3** does not correspond to all the patients, as many patients who had similar responses are grouped into single graph lines for the sake of clarity.

### Relationship between the amount of pacing and changes in blood pressure

**Figure 4:**
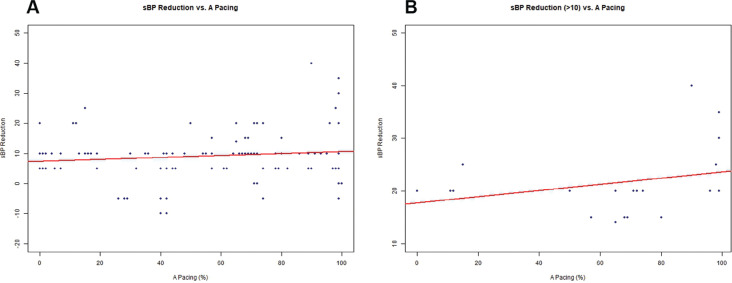
**A:** Plot describing the correlation between reduction in systolic blood pressure and percent atrial pacing among responders. The positive slope does not reach statistical significance (*R*^2^ = 0.022; *P* = .09). **B:** A more robust correlation (still statistically not significant) between systolic blood pressure reduction and percent atrial pacing when the blood pressure decline is stratified at a decline of >10 mmHg (*R*^2^ = 0.086; *P* = .19) is displayed. *Abbreviation:* sBP, systolic blood pressure.

**Figure 5:**
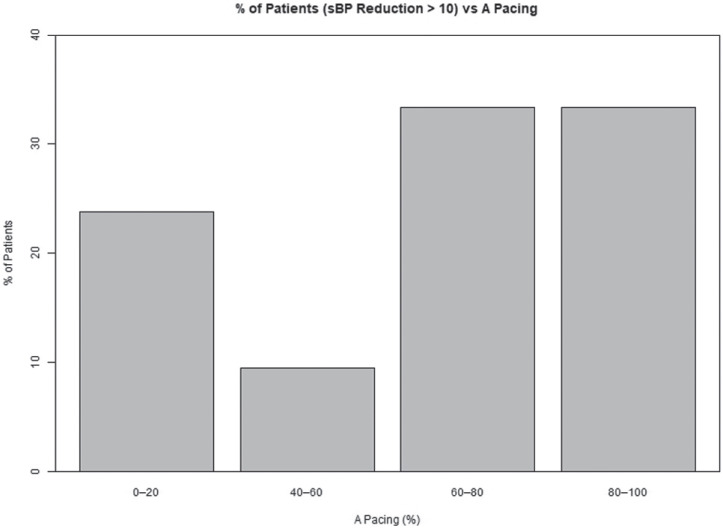
Distribution of atrial pacing among patients who experienced a >10-mmHg decline in sBP. The group of responders who manifested a reduction in sBP of >10 mmHg (n = 21) is divided into bins of percentage of atrial pacing. As illustrated, most of the patients with this response in sBP were found within the two pacing bins that represent >60% atrial pacing. *Abbreviation:* A-pacing, atrial pacing.

We sought a statistical correlation between the amount of pacing in each chamber and changes in sBP **(Figure 4)**. Using a linear regression model, correlation between atrial pacing and reduction in sBP among the responders revealed a progressive decline in sBP with an increased percentage of atrial pacing, but this did not reach statistical significance (*R*^2^ = 0.022; *P* = .09). An analysis of responders who experienced an sBP decline of >10 mmHg (n = 21) showed a stronger correlation with atrial pacing, albeit without reaching statistical significance (*R*^2^ = 0.09; *P* = .19; Pearson correlation coefficient = 0.3). A more useful presentation of the data is potentially provided in **Figure 5**, wherein patients are presented as bins of percentage of atrial pacing among responders with an sBP decline of >10 mmHg. As illustrated, most of the patients with this response in sBP were found within the two pacing bins that represent >60% atrial pacing. A negative correlation (statistically not significant) was found between right ventricular (RV) pacing and a decline in sBP, ie, there were fewer sBP changes with increasing RV pacing (Pearson correlation coefficient = −0.09). Similarly, increased atrial pacing supported a greater decline in dBP (*R*^2^ = 0.04; *P* = .02; Pearson correlation coefficient = 0.2), which did not quite reach statistical significance. Increasing RV pacing was again negatively correlated with a decline in dBP (Pearson correlation coefficient = −0.22).

### Stratifying blood pressure responses based on pacing percentage in each chamber

Based on prior published studies in patients with permanent RV pacing,^19,20^ we stratified responders into groups of >40% and <40% RV pacing. **Table 3** illustrates the results. The optimal BP response was seen amongst the 45 patients who paced ≥50% in the right atrium (RA) and <40% in the right ventricle (with declines of 12 mmHg in sBP, 6 mmHg in dBP, and 1.62 in the number of medications, respectively). The results were similar when the RV pacing was dichotomized at 50% (48 patients experienced reductions of 11 mmHg, 6 mmHg, and 1.52 in sBP, dBP, and medications, respectively). For the entire study population (N = 176), stratification according to atrial and ventricular pacing percentages, as stated previously, yielded results of −11 mmHg (sBP), −6 mmHg (dBP), and −1.55 medications in those who were paced >50% in the RA and <40% in the right ventricle.

### Indications for pacemaker implantation

As indicated in **Table 1**, responders were more likely to have a sinus node disease indication for pacing, but this did not reach statistical significance. Pacing indication did not prove to be a statistically significant predictor of BP response to long-term pacing.

### Left ventricular ejection fraction

No significant differences were found between the two groups of patients prior to PP. LVEF was not significantly affected by PP in either group **(Table 1)**.

## Discussion

The results of this study support and extend prior observations that PP in elderly patients with preserved LVEF and DRH results in significant improvements in BP control. The important findings in this study include the following observations: (1) a statistically significant number of elderly patients with DRH showed a significant decline in sBP following the implementation of PP; (2) the magnitude of the improvement in sBP (9 mmHg for the entire responder group) is significant and encouraging; (3) the improvements in sBP and dBP are particularly notable in the subgroup of patients in whom atrial pacing exceeds 50% and ventricular pacing is <40%, where reductions of 12 and 6 mmHg are seen, respectively; (4) the pacing-related decline in sBP appears to be directly related to the amount of atrial pacing, and while the correlation did not reach statistical significance, most of the responders who achieved a >10-mmHg improvement in sBP were in the group that paced >60% in the atrium; and (5) LVEF was similar in both groups and did not significantly change after pacing in either group.

The effects of neuromodulation on HTN have been the subject of extensive investigation over the past few decades. Initial studies on baroreflex activation therapy devices, endovascular baroreflex amplification therapy, carotid body catheter ablation, and renal artery denervation via catheter ablation have shown favorable results in lowering BP.^8,10–17^ However, larger randomized controlled trials are undoubtedly needed to validate invasive procedures on the carotid arteries, and the results of randomized placebo-controlled trials of renal artery denervation have to date shown only a modest (4/2 mmHg) magnitude of benefit.^21^

Of greater relevance to this study is the published observation that pacemaker-based cardiac neuromodulation therapy (CNT) is feasible and acceptably safe in patients with HTN and an indication for pacemaker implantation.^8,9^ This finding is particularly interesting because ∼66% of the patients who require pacemaker therapy have arterial HTN.^8^ The initial unblinded studies of CNT employing a sequence of variably timed shorter and longer AV intervals showed a decrease in BP levels in pacemaker patients with DRH.^9^ The effects of CNT on BP were confirmed by a double-blind randomized pilot study,^9^ whose authors suggested that a combination of decreased ventricular preload and a putative modulation of the autonomic nervous system might prevent baroreceptor-based sympathetic activation and serve as a potential mechanism of the observed BP reduction. However, the results of these studies have also raised questions about the long-term safety and quality of life in patients undergoing pacing with short, programmed AV delays.^22^ Specifically, it is well known that chronic RV pacing can increase the risk of heart failure,^19,20^ while long-term pacing with programmed short AV delays can cause “pacemaker syndrome” and various atrial arrhythmias.^22^ Both are crucial features of the proposed CNT pacing algorithm. In contrast, our study was a retrospective analysis of patients undergoing PP with conventional clinically indicated programming of dual-chamber pacemakers, ie, minimization of RV pacing.

This retrospective study, by definition, was not designed to elucidate physiologic mechanisms. However, given the significant changes in BP seen in our hypertensive patient population following the implantation of permanent pacemakers, we propose two possible physiologic mechanisms. First, it has long been known that sympathetic nerve activity (SNA) declines with dual-chamber pacing among patients with normal LVEFs at clinically relevant pacing rates.^23^ Intriguingly, the BP-lowering effect of an increased atrial pacing rate in an office-based pacing study is blunted in patients taking β-blocking medications,^24^ suggesting a blockade of pacing-induced lowering of SNA. Further insight might be provided by a study in which ambulatory SNA is measured in a similar patient population.

Second, it is known that atrial pacing in an experimental animal model is associated with a significant release of endogenous atrial natriuretic peptide.^25^ While brain natriuretic peptide (BNP) levels have generally been thought to indicate worsening of LV function, elevated levels are associated with RV pacing and lessened by dual-chamber pacing^26–30^; nonetheless, it may be the case that the increased atrial pacing seen in this study’s responders may have engendered BNP release, which may have played a role in the improved control of BP in these patients. As BNP levels were not available for most of this study’s patients, this remains, of course, a matter of conjecture that is yet to be confirmed.

The relationship between cardiac pacing and BP control requires larger studies with longer follow-up to confirm the long-term safety and efficacy in lowering BP, prior to entering clinical practice.^31^

### Limitations

The main limitation of the present study is its retrospective and non-randomized study design. The acceptable safety profile and strong efficacy observed in the present study support the completion of further prospective randomized studies.

## Conclusions

This relatively large retrospective long-term study provides initial evidence that HTN treatment with a conventional dual-chamber pacemaker-based device appears safe and effective at intermediate and long-term follow-up. The current study included patients with DRH who have standard indications for PP. The significant reduction in sBP and dBP is more closely related to atrial pacing, in contrast to previously published studies in patients with DRH and PP, as discussed. If validated, a clear advantage of using BP as an endpoint for adjusting atrial pacing becomes self-evident. Factors requiring further clarification in longer-term randomized studies include assessments of safety (impact on LV size and function, atrial size, and arrhythmias), the impact on BNP, and autonomic nervous system activity. With further proof of safety and efficacy, pacing therapy for HTN may yet be a new indication for PP in the appropriate patient population.

## Figures and Tables

**Table 1:** Characteristics of the Study Patients

	Responders n = 126	Non-responders n = 50	*P* Value
Age (years), mean (±SD)	75.2 (6.4)	73.7 (8.6)	.7
Male sex, %	76.2	82	.5
Pacing indication SSS, %	61.9	46	.06
Pacing indication AVB, %	50.8	72	.01
A pace (%), mean (±SD)	50 (30)	30 (40)	.002
V pace (%), mean (±SD)	40 (40)	60 (40)	.006
FU (months), mean (±SD)	75.5 (55.5)	74.3 (63.2)	.5
Pre-sBP (mmHg), mean (±SD)	130.2 (9.8)	121.5 (6.6)	<.01
Pre-dBP (mmHg), mean (±SD)	79 (5.8)	79.5 (7.5)	.6
Post-sBP (mmHg), mean (±SD)	121.3 (10.2)	126.3 (6.7)	.001
Post-dBP (mmHg), mean (±SD)	75.9 (7.5)	78.1 (7.3)	.09
Pre-#meds, mean (±SD)	3.3 (0.5)	3 (0.1)	<.001^a^
Post-#meds, mean (±SD)	2.7 (1)	3.2 (0.5)	<.01^b^
Pre-EF (%), mean (±SD)	54.6 (3.6)	56 (2.7)	.01
Post-EF (%), mean (±SD)	56.1 (3.1)	55.8 (3.6)	.7
Medications			
Diuretics, %	81.7	80	.8
BB, %	57.9	68	.2
CCB, %	61.1	30	<.01
ACEI/ARB, %	94.4	100	.2
α-Blockers, %	11.9	18	.3
ALD, %	19.8	10	.2

*Abbreviations:* A pace, atrial pacing; ACEI, ACE inhibitor; ALD, aldosterone antagonist; ARB, angiotensin receptor blocker; AVB, atrioventricular block; BB, β-blocker; CCB, calcium channel blocker; dBP, diastolic blood pressure; EF, ejection fraction; FU, follow-up; meds, medications; sBP, systolic blood pressure; SD, standard deviation; SSS, sick sinus syndrome; V pace, ventricular pacing. ^a^The highlighted comparison with corresponding *P* value is a comparison of numbers of hypertension medications between before and after permanent pacing, using a paired *t* test (3.3 vs. 2.7). ^b^For responders, there is a significant increase in the number of medications after permanent pacing (3 vs. 3.2).

**Table 2:** Blood Pressure Before and After Pacing for Responders and Non-responders

		Pre-pacing Mean (±SD)	Post-pacing Mean (±SD)	Difference in Mean (mmHg)	*P* Value
Responders	sBP	130 (9.8)	121 (10.2)	9	<.01
	dBP	79 (5.8)	76 (7.5)	3	<.01
Non-responders	sBP	122 (6.6)	126 (6.7)	−4	<.01
	dBP	80 (7.5)	78 (7.3)	2	.3

*Abbreviations:* dBP, diastolic blood pressure; sBP, systolic blood pressure; SD, standard deviation.

**Table 3:** Changes in Blood Pressure and Number of Medications Stratified by Atrial (50%) and Ventricular (40%) Pacing Among Responders

A Pacing	V Pacing	n	Mean (±SD)		
			Change in sBP (mmHg)	Change in dBP (mmHg)	Change in the Number of Medications
<50%	<40%	29	−5.00 (7.3)	−1.55 (7.8)	−0.45 (0.8)
<50%	≥40%	34	−9.12 (5.1)	−3.82 (8)	0.21 (0.5)
≥50%	<40%	45	−12.00 (9.2)	−6.33 (7.9)	−1.62 (1)
≥50%	≥40%	18	−7.17 (2.9)	3.33 (6.4)	0.22 (0.7)

*Abbreviations:* A pacing, atrial pacing; dBP, diastolic blood pressure; sBP, systolic blood pressure; SD, standard deviation; V pacing, ventricular pacing.
